# Synthesis and Characterization of Fully Bio-Based Butylene Succinate Oligomers with Varying Molecular Weights for Sustainable Food Packaging Applications

**DOI:** 10.3390/polym17091276

**Published:** 2025-05-07

**Authors:** Carmen Olivas-Alonso, Yaiza Flores, Antxon Martínez de Ilarduya, Amparo Chiralt, Sergio Torres-Giner

**Affiliations:** 1 University Institute of Food Engineering—FoodUPV, Universitat Politècnica de València (UPV), Camino de Vera s/n, 46022 Valencia, Spain; carolal1@etsii.upv.es (C.O.-A.); yflofer@upvnet.upv.es (Y.F.); dchiralt@tal.upv.es (A.C.); 2 ETSEIB, Chemical Engineering Department, Universitat Politècnica de Catalunya · BarcelonaTech, Diagonal 647, Planta G 1E, 08028 Barcelona, Spain; antxon.martinez.de.ilarduia@upc.edu

**Keywords:** oligomer, butylene succinate, biopolymer formulations, food packaging

## Abstract

The development of bio-based and biodegradable materials is critical for reducing environmental impact and addressing global challenges associated with the extensive use of plastics in packaging applications. In this study, linear oligomers of butylene succinate (OBS) with three different molecular weights were synthesized using succinic acid (SA) and 1,4-butanediol (BDO), both monomers derived from biomass. The synthesized fully bio-based OBS samples were characterized in terms of their molecular structure, degree of polymerization, crystallinity, and thermal properties, showcasing their potential as additives for biopolymers in food packaging. Oligomers with weight-average molecular weight (M_w_) values of 2050 g·mol^−1^ (OBS-L), 16,150 g·mol^−1^ (OBS-M), and 33,147 g·mol^−1^ (OBS-H), and Ð values in the 1.7–1.8 range were successfully synthesized. The results showed that the thermal degradation stability of OBS slightly increased, while the crystallinity decreased with increasing molecular weight. Furthermore, the analysis of the evolution of the lattice parameters suggested that oligomers with shorter chains favored crystal organization, resulting in a crystal unit cell with denser packing.

## 1. Introduction

The development of bio-based and biodegradable materials is crucial for mitigating environmental impacts and addressing the global challenges associated with the use of non-renewable resources and non-biodegradable products in food packaging applications [1]. In this context, poly(butylene succinate) (PBS) has emerged as a promising aliphatic biopolyester with a high potential to replace polyolefins since it combines good mechanical, thermal, and barrier properties and can be processed with conventional plastic equipment [2]. In particular, fully bio-based PBS, which is synthesized from renewable succinic acid (SA) and 1,4-butanediol (BDO), has gained significant attention due to its potential to minimize the carbon footprint and promote environmental sustainability [3]. However, the processing and performance of most PBS grades still need to be modulated for certain applications, such as flexible and barrier packaging for food [4].

Similar to other polymers, the final PBS properties depend not only on its inherent characteristics but also on its processing methods, modifications, and incorporated additives [5,6]. Thus, the use of additives can further modulate the polymer matrix properties and facilitate the processing of thermoplastic materials, enhancing product versatility and extending their applications [7,8]. In particular, oligomers have demonstrated considerable potential for application as polymer additives. For instance, Yin et al. [9] synthesized fully bio-based glycerol succinate oligoesters using a solvent-free method, which were subsequently melt-blended with poly(ε-caprolactone) (PCL) to tailor PCL’s mechanical properties and degradation rate without compromising its processability or strength. It was observed that oligoesters were well-dispersed within the PCL matrix, influencing crystallization and significantly accelerating degradation. Notably, following this oligomer blending strategy, there was no substantial decrease in mechanical performance. In another study, Bernabé et al. [10] examined the properties of poly(3-hydroxybutyrate-*co*-3-hydroxyvalerate) (PHBV) blends containing oligomers of lactic acid (OLA) and their performance during mechanical recycling. The authors found that OLA acted as a plasticizer, enhancing the processability and ductility. However, the oligomer also partially degraded or migrated during repeated processing. Consequently, the resultant reprocessed material exhibited higher stiffness and lower elongation, highlighting the plasticizer loss and its influence on the final properties.

One of the most interesting uses of oligomers as polymer additives is their incorporation into their respective polymer matrices, as compatibility is a critical parameter in polymer blends and formulations. In particular, oligomers are expected to act as molecular mobility enhancers in polymers, directly influencing their final properties [11]. In this regard, Niiyama et al. [12] blended high- and low-molecular-weight PCL to enable controlled and accelerated degradation. This approach facilitated the processability of the biopolymer, decreased its crystallinity, and increased its hydrophilicity. Their single-polymer strategy offers a simple solution for tailoring scaffold lifetimes for biomedical applications. In another study, Burgos et al. [13] synthesized OLAs with two different molecular weights (670 and 1000 Da) to serve as plasticizers for polylactide (PLA). The addition of OLA significantly increased PLA flexibility and elongation while maintaining transparency and yielding biodegradable flexible films for food packaging. The high miscibility between PLA and OLA was confirmed by the observation of a single glass transition temperature (T_g_). Similarly, Ambrosio-Martín et al. [14] incorporated OLAs into PLA, reporting an anti-plasticizing effect that reduced the free volume of the biopolymer and decreased its oxygen and water permeabilities while preserving its good processability. A similar effect was observed after incorporating two different commercial OLA grades into PLA composites with spent coffee grounds (SCGs) [15]. The simultaneous addition of the two OLAs improved the tensile strength of the green composite by nearly 36% and 60%, respectively. Higher performance was observed when OLA functionalized with maleic anhydride groups was used, which was ascribed to the chemical interaction achieved between the biopolyester and the lignocellulosic fillers.

In this regard, oligomers of butylene succinate or oligo(butylene succinate) (OBS) have been reported to be obtained only during the first steps of the PBS polymerization process [8,16,17,18] or even produced after the disintegration or depolymerization of the biopolyester [19]. To date, various parameters have been described to affect the rate of reactions and the subsequent molecular weight of OBS, such as the type of solvent, catalyst, and energy source. For example, Rajgond et al. [20] explored the use of solvents to extract OBS after synthesizing PBS. The authors observed that, in the case of diethyl ether, an oligomer with a molecular weight of 10,000 g⋅mol^−1^ was achieved, in contrast to the oligomers with lower molecular weight values (less than 4400 g⋅mol^−1^) obtained using other solvents. In another study, Chen et al. [21] synthesized telechelic PBS oligomers with different molecular weights by introducing 2-ureido-4[1H]-pirimidinona (UPy) units to the chain ends, and their crystallization behaviors—especially nucleation processes—were studied in detail. Thus, a series of these OBS with varying degrees of polymerization were synthesized from 1000 g⋅mol^−1^ to approximately 4000 g⋅mol^−1^, and it was observed that UPy stacks formed via quadruple hydrogen bonding suppressed the crystallization rate in comparison with the oligomers with hydroxyl (–OH) end groups. Moreover, investigation of the nucleation behavior revealed that low-molecular-weight oligomers showed reduced growth and nucleation abilities due to the confinement effect of the UPy stacks, whereas as their molecular weight increased, the nucleation ability was strengthened, and consequently, the nucleation density increased.

Despite these previous reports and advances, significant knowledge gaps remain in the synthesis and characterization of OBS. In particular, further research is needed to elucidate how molecular weight can affect both the chemical and physical properties of OBS, such as crystallinity, glass transitions, and thermal stability. In the present study, fully bio-based OBS with different molecular weights were synthesized from renewable SA and BDO by controlling the conditions and extension of a previously developed multi-step polycondensation process. The resultant oligomers were characterized in terms of their molecular structure, crystallinity, and thermal properties to evaluate their potential as novel additives in biopolyester-based food packaging materials.

## 2. Materials and Methods

### 2.1. Materials

Succinic acid was obtained by gas-phase oxidation of furfural derived from biomass (corn cobs). Thereafter, BDO was produced by high-pressure catalytic hydrogenation of the previously obtained renewable succinic acid. The detailed production of both monomers has been reported in our previous study [16]. The catalyst titanium (IV) isopropoxide (TTiP) with 97% purity was obtained from Sigma-Aldrich S.A. (Madrid, Spain) in extra-dry toluene (max. 0.005% H_2_O with molecular sieves (Scharlab S.A., Barcelona, Spain).

### 2.2. Oligomerization

**Scheme 1:**
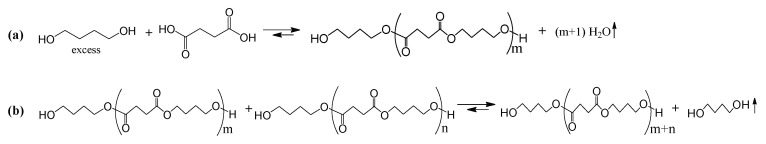
(**a**) Esterification and (**b**) transesterification reactions during the synthesis of oligo(butylene succinate) (OBS).

As shown in Scheme 1, OBSs were synthesized by polycondensation reaction of renewable SA with BDO. Oligomerization was performed in two steps: esterification to produce short-chain oligomers (Scheme 1a), followed by catalytic transesterification under controlled conditions to increase their molecular weight (Scheme 1b). The reaction was conducted using an excess of BDO, which contributed to the formation of oligomers with –OH end groups as the main products.

**Figure 1:**
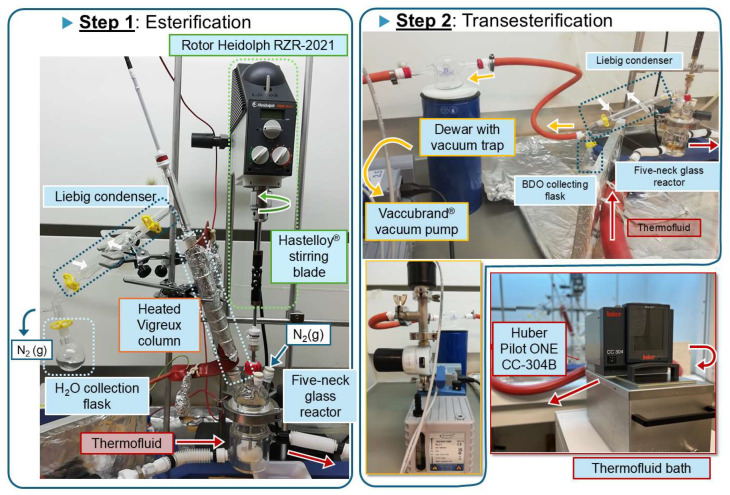
Oligomerization unit with different elements involved during the esterification step (**left**) and transesterification step (**right**).

The reaction was carried out in a 100-mL glass-jacketed reactor with five necks, as shown in Figure 1. The reactor was connected to a Huber Pilot Pilot ONE CC-304B immersion bath (Peter Huber Kältemaschinenbau AG, Offenburg, Germany) for precise temperature control. A mechanical stirrer (Heidolph RZR-2021 rotor, Heidolph Instruments GmbH & Co. KG, Schwabach, Germany) and a Vigreux condenser column with a glass distillation adapter were connected through five necks. Further details of this oligomerization/polymerization unit can be found in the literature [22].

Before starting the reaction, renewable SA was placed in the reactor, and the system was purged for 30 min with a constant nitrogen flow-rate of 200 mL·min^−1^ at room temperature to remove oxygen and moisture from the reactor headspace. After purging, the system was maintained under an inert atmosphere and positive pressure (0.2 mL·min^−1^).

#### 2.2.1. Esterification

The esterification reaction was then carried out in two consecutive stages by the reaction of SA with excess BDO, as shown in Scheme 1a. Firstly, BDO was added dropwise to the reactor with SA at a 1:1.5 diacid-to-diol molar ratio in order to maximize the formation of –OH end groups in OBS [23,24]. During the first step of the oligomerization process, stirring was set at 50 rpm, and the temperature was raised to 180 °C at a heating rate of 3.3 °C·min^−1^. The water vapor formed during the esterification reaction was removed by flowing a nitrogen stream (0.2 mL·min^−1^) and collected in a Liebig condenser. In the heated Vigreux column, BDO was separated from water and recovered in the reactor vessel at a temperature of 130 °C. The first stage of the esterification reaction was carried out for approximately 3 h, until no more water was formed. Subsequently, the experimental setup was modified in order to remove excess BDO from the reactor mass. To this end, during the second stage, the temperature was reduced to room temperature, and the Vigreux column was replaced with a Liebig condenser connected to a high-vacuum pump (Vacuum pump RZ 2.5, Vacuu-Select^®^, Vacuubrand GmbH, Wertheim, Germany). The temperature was then increased to 180 °C at a rate of 3.3 °C·min^−1,^ and the pressure was gradually reduced to 300 mbar, while the stirring was maintained at 50 rpm. This step was performed for at least 1 h until no more BDO was collected. No further purification was performed. To conclude this step, the reactor system was cooled to room temperature, and a white solid was obtained. The oligomer sample resulting from this process is referred to as OBS-L due to its expected low molecular weight.

#### 2.2.2. Transesterification

The transesterification reaction of the short-chain oligomers obtained during the previous esterification step resulted in the formation of OBS samples with higher molecular weights. This reaction is mainly based on the reaction of the terminal –OH groups of some oligomers with the ester groups of other oligomers (Scheme 1b). BDO was released during the oligomer chain growth. The reorganization and exchange of ester groups between oligomer chains were driven by heat, high vacuum, and a metallic catalyst (TTiP). The same experimental setup used during the last stage of the esterification step was maintained. Briefly, the oligomer was first melted by reaching a temperature of approximately 120 °C. Then, the catalyst, a 5 wt. % solution of TTiP, was added in the form of a suspension through one of the septum lids using a glass syringe. Thereafter, the temperature of the system was raised to 230 °C at 2 °C·min^−1,^ and vacuum was progressively applied to avoid oligomer sublimation until achieving a <1 mbar of pressure, keeping 50 rpm of stirring. Chain growth by transesterification was performed for 1 h (OBS-M) and 2 h (OBS-H) to produce oligomers with medium and high molecular weights, respectively. Once oligomerization was complete, the reactor system was cooled to room temperature.

Finally, all samples were milled into powder using an IKA^®^ M20 basic analytical mill (IKA^®^-Werke GmbH & Co. KG, Staufen, Germany) and stored unwashed at room temperature at 0% relative humidity (HR) in phosphorous pentoxide (P_2_O5, Sigma-Aldrich S.A.). The three synthesized oligomers, OBS-L, OBS-M, and OBS-H, were characterized in terms of their molecular weight, thermal properties, and crystallinity.

### 2.3. Chemical Characterization

#### 2.3.1. Nuclear Magnetic Resonance Spectroscopy

Proton nuclear magnetic resonance (^1^H-NMR) was used as a quantitative technique for end-group analysis and determination of the number-average molecular weight (M_n_) of OBS. ^1^H-NMR spectra were recorded on a Bruker AMX-300 spectrometer (Bruker Corporation, Billerica, MA, USA) at 25 °C, operating at 300.1 MHz. Approximately 10 mg of the sample was dissolved in 1 mL of deuterated chloroform (CDCl_3_, EuroIsotop, Saint Aubin, France) using tetramethylsilane (TMS, EuroIsotop) as an internal reference. A total of 320 scans were obtained. To determine the M_n_ values, a drop of trifluoroacetyl anhydride (Merck Life Science S.L.U., Madrid, Spain) was added to the NMR tube to downfield shift the signal due to CH_2_COOH. The signals from the inner COOCH_2_ and CH_2_OH/CH_2_COOH end groups were integrated to determine the degree of oligomerization. The spectra were processed and analyzed using Bruker 1D WIN NMR computer software.

#### 2.3.2. Gel Permeation Chromatography

The values of M_n_, weight-average molecular weight (M_w_), and dispersity (Ð) of the synthesized oligomers were determined by gel permeation chromatography (GPC) using a Waters GPC instrument equipped with refractive index (RI) and ultraviolet (UV) detectors. Between 2 and 3 mg of the sample was dissolved in 100 µL of chloroform (CHCl_3_, HPLC grade, Fisher Scientific S.L., Alcobendas, Spain) and filtered using a 0.22-µm PTFE filter (Phenomenex, Alcobendas, Spain). Then, 100 mL of this solution was injected into the system and eluted with CHCl_3_ at a flow-rate of 0.5 mL·min^−1^. HR5E and HR2 Waters linear Styragel columns (7.8 mm × 300 mm, pore 10^3^–10^4^ Å, Waters Cromatografía, S.A, Cerdañola del Vallés, Spain) packed with cross-linked polystyrene (PS) and protected with a precolumn were used. PS standards with narrow molecular weight ranges were employed to generate the calibration curve. The molecular weight distribution (MWD) was calculated from the signal heights in the elugrams by the application of the slice method [25,26]. Thus, the M_n_ and M_w_ values were obtained from Equations (1) and (2), respectively:

(1) $$M_{n}=\frac{\sum_{i=1}^{N}h_{i}}{\sum_{i=1}^{n}\left(h_{i}∕M_{i}\right)}$$

(2) $$M_{w}=\frac{\sum_{i=1}^{N}\left(h_{i}{\cdot M}_{i}\right)}{\sum_{i=1}^{N}h_{i}}$$

where *h_i_* corresponds to the concentration signal, and *M_i_* is the molecular weight of each slice (*i*).

### 2.4. Physical Characterization

#### 2.4.1. Thermogravimetric Analysis

The thermal stability was determined by TGA using a thermogravimetric analyzer (TGA 1 Stare System analyzer, Mettler-Toledo GmbH, Greifensee, Switzerland) in the thermal range from 25 °C to 700 °C at a heating rate of 10 °C·min^−1^. The TGA and first-derivative thermogravimetric analysis (DTGA) curves were obtained using STARe Evaluation Software (Mettler-Toledo, GmbH) to obtain the onset degradation temperature (T_onset_), temperature of the maximum degradation rate (T_peak_ in DTGA curves), and end degradation temperature (T_end_).

#### 2.4.2. Differential Scanning Calorimetry

The phase transitions of the oligomer samples were analyzed using differential scanning calorimetry (DSC) in a DSC823^e^ Star^e^ (Mettler-Toledo GmbH), operating under a nitrogen atmosphere (10 mL·min^−1^). Samples (5–6 mg) were placed in aluminum pans and tightly sealed. These were then placed in the calorimeter, and the following three-step program was applied: first heating from −40 to 150 °C to erase the thermal history that the oligomers may have experienced during their synthesis, followed by cooling to −40 °C, and then a second heating to 150 °C. An empty aluminum pan was used as a reference sample. Each sample was analyzed in duplicate using heating and cooling rates of 10 °C·min^−1^. The degree of crystallinity (*χ*_c_) was calculated by means of Equation (3):

(3) $$\chi_{c}=\frac{{∆H}_{m}-{∆H}_{cc}}{{∆H{}^{\circ}}_{m}}\cdot 100$$

where *ΔH_m_* and *ΔH_cc_* correspond to the melting and cold crystallization enthalpies of the oligomers, respectively, both obtained from the second heating scan. Regarding the theoretical melting enthalpy of a fully crystalline OBS (*ΔH°_m_*), different values reported for PBS were used: 110.3 J/g [27], 102 J/g [28,29], and 200 J/g [30,31].

#### 2.4.3. X-Ray Diffraction Analysis

The crystallinity of the OBS samples was also studied using an X-ray diffractometer (AXS/D8 Advance, Bruker AXS Advanced X-ray Solutions GmbH, Karlsruhe, Germany). Diffractograms were obtained at 40 kV and 40 mA, with a step size of 0.04°·min^−1^ and a 2Ө scanning angle between 5° and 50°. The powder of the oligomer samples, previously conditioned at 0% RH, was compactly placed in the holder of the XDR equipment. Data were obtained using a 1D LynxEye detector with XRD Commander software (Bruker AXS Advanced X-ray Solutions GmbH) and processed with DIFFRAC.EVA (Bruker AXS Advanced X-ray Solutions GmbH ) and DRXWin (Windows, version 2.3) software. The values of *χ*_c_ were calculated using Equation (4) by deconvolution of the amorphous halo using the Origin software (OriginLab Corporation, Northampton, MA, USA) [32]:

(4) $$\chi_{c}=\left(\frac{A_{c}}{A_{c}+A_{a}}\right)\cdot 100$$

where *A_c_* and *A_a_* represent the crystalline and amorphous areas, respectively.

The lattice parameters (a,b,and c) and the monoclinic angle (β) were calculated considering that oligomers exhibit diffraction peaks corresponding to the monoclinic crystal lattice of PBS in the α-form [33]. Bragg’s Law (Equation (5)) was applied to obtain the values of interplanar spacing *(d_hkl_*), where n=1 is the order of diffraction, λ is the wavelength of the incident radiation (1.542 Å for CuK_α_ radiation), and *θ* is the angles between the incident X-ray beam and each respective crystal plane:

(5) $$n\cdot \lambda =2\;{\cdot d}_{hkl}\cdot \sin(\theta )$$

Thereafter, Equation (6) was used as the general equation in a monoclinic crystal system to determine the lattice parameters and angles for each interplanar distance [34]:

(6) $$\frac{1}{{d^{2}}_{hkl}}=\frac{h^{2}}{a^{2}\cdot {sin}^{2}\beta }+\frac{k^{2}}{b^{2}}+\frac{l^{2}}{c^{2}\cdot {sin}^{2}\beta }-\frac{2\cdot h\cdot l\cdot cos\beta }{a\cdot c\cdot {sin}^{2}\beta }$$

where h,k, and l are the Miller indices for each specific diffraction peak. Thus, a system of nonlinear equations was constructed from the *d_hkl_* values of the first four characteristic diffraction peaks. A Python code (Python version 3.11.10) was developed using the NumPy library to solve the system of equations based on the input of the four interplanar distances associated with the four main crystalline peaks in the XRD analysis.

### 2.5. Statistical Analysis

Results were submitted to analysis of variance (ANOVA) using Statgraphics Centurion XVII-64 software (Manugistics Corporation, Rockville, MD, USA). Significant differences were considered with a significance level greater than 95% (*p* < 0.05).

## 3. Results and Discussion

### 3.1. Molecular Weight of Oligomers

**Figure 2:**
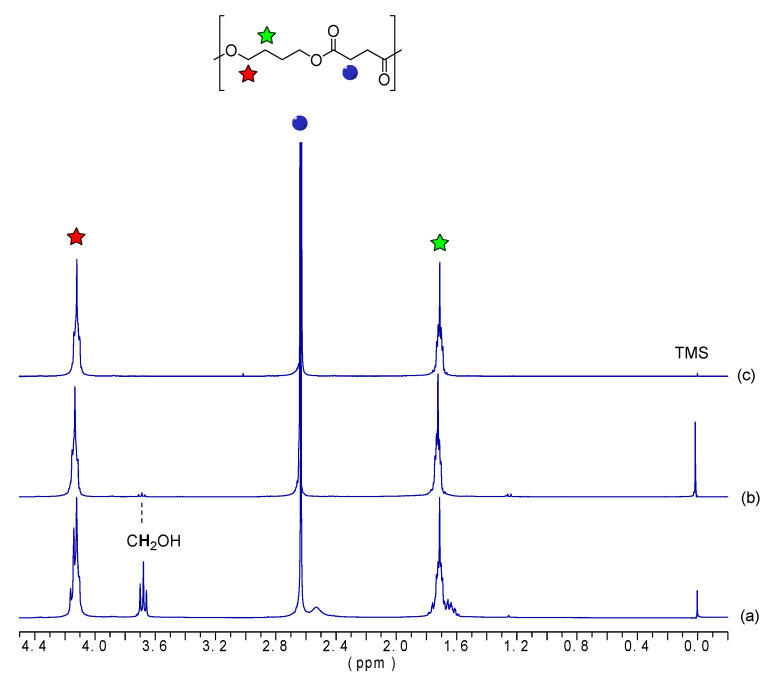
Proton nuclear magnetic resonance (^1^H-NMR) spectra with peak assignments of oligomers of butylene succinate (OBS) obtained at the end of the first esterification step (**a**) and after 1 h (**b**) and 2 h (**c**) of the transesterification step.

**Figure 3:**
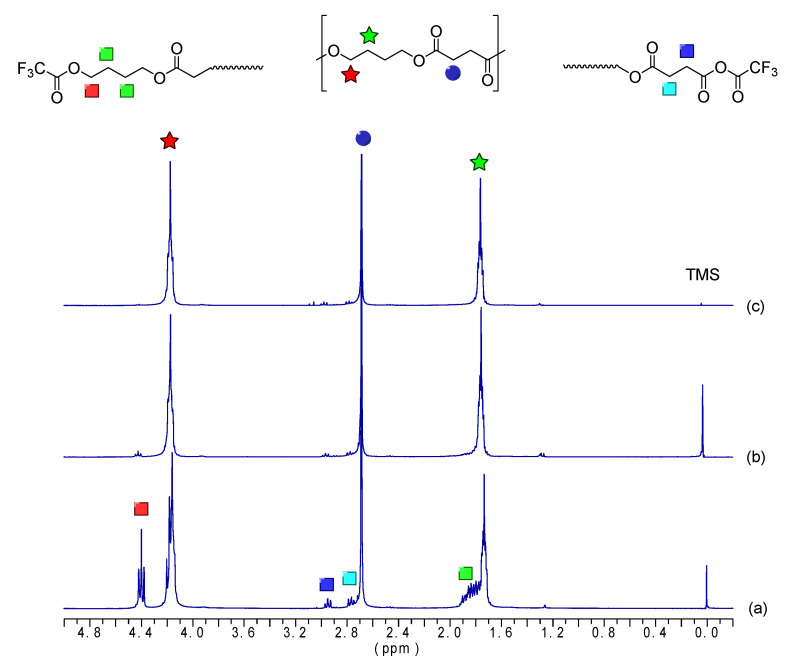
Proton nuclear magnetic resonance (^1^H-NMR) spectra with peak assignments of oligomers of butylene succinate (OBS) obtained at the end of the first esterification step (**a**) and after 1 h (**b**) and 2 h (**c**) of the transesterification step, after sample derivatization with trifluoroacetic anhydride to obtain the carboxyl end groups.

The chemical structure of the obtained oligomers were analyzed using ^1^H-NMR. Figure 2 displays the ^1^H-NMR spectra with peak assignments for the oligoesters. Figure 2a shows the spectra of the oligomer obtained at the end of the esterification step (OBS-L), whereas Figure 2b,c show the spectra after the transesterification step for times of 1 h (OBS-M) and 2 h (OBS-H), respectively. The two broad triplets appearing at 4.1 and 1.7 ppm can be assigned to the first and second methylene of the oxybutylene units, respectively [35,36], whereas the two methylenes of the succinate unit appeared as a singlet at 2.6 ppm. This agrees with the findings reported by Ding et al. [37], with peaks at 4.28 and 1.82 ppm assigned to the methylene protons of the BDO units, while the peak at 2.84 ppm was assigned to the methylene protons in SA units. Moreover, it can be observed that the signal corresponding to the CH_2_OH end groups, appearing at 3.7 ppm, decreased in intensity as the oligomerization progressed. This signal and the one due to the CH_2_COOH end-group of the samples derivatized with trifluoroacetic anhydride (Figure 3), observed at 2.8 ppm, were used to determine the M_n_ values of the oligomers [38,39], which were calculated from the integrals displayed in Table 1 and are gathered in Table 2.

**Figure 4:**
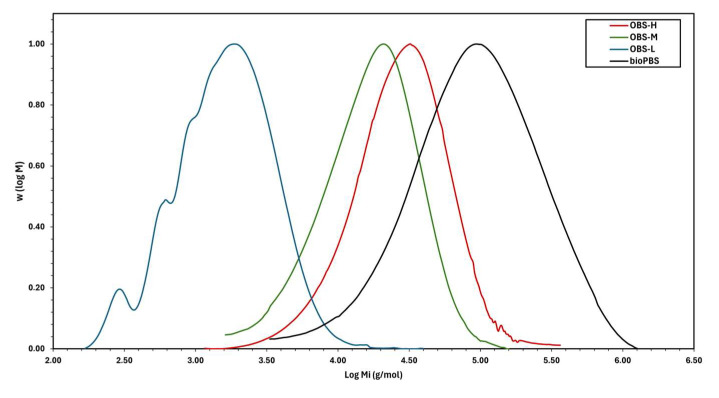
Molecular weight distributions of oligo(butylene succinate) with high (OBS-H), medium (OBS-M), and low molecular weights (OBS-L).

The molecular weights of the synthesized oligomers were also determined by GPC. In Figure 4, the ratio of the average molecular weight in the log scale [*W* (*log M_i_*)] is represented as a function of the number-average molecular weight in the log scale (*log M_i_*). These data were obtained from the signal heights and retention times in GPC elugrams. These plots represent the MWD of the three oligomers, which in all cases exhibited a monomodal and moderately symmetrical distribution with different average values of molecular weight. Table 2 also includes the values of both M_n_ and M_w_ obtained from the GPC measurements, confirming that the three oligomers have different molecular weights but similar dispersity. As expected, the OBS-H sample, which underwent the longest transesterification time (2 h), reached the highest molecular weight (M_n_ = 18,650 g·mol^−1^ and M_w_ = 33,147 g·mol^−1^), reflecting the effect of the longer reaction time on chain growth. Thus, OBS-L (M_n_ = 1150 g·mol^−1^ and M_w_ = 2100 g·mol^−1^) without transesterification and OBS-M (M_n_ = 8700 g·mol^−1^ and M_w_ = 16,150 g·mol^−1^) with a shorter transesterification time showed lower molecular weight values than OBS-H. Therefore, the transesterification step notably promoted chain growth, whereas smaller differences were observed between the oligomers produced after 1 h and 2 h of transesterification. Furthermore, the molecular weight values of both OBS-H and OBS-M were close to that of PBS, indicating that these samples may also be classified as either biopolyesters of low molecular weight or oligoesters of high molecular weight.

The molecular weights of these oligomers are in the range of those of other OBS reported previously. For instance, Papaspyrides et al. [40] synthesized OBS rich in –OH end groups using an excess of BDO (diol-to-diacid molar ratio of 1.25:1.00), which was thereafter applied for solid-state polymerization (SSP) to yield PBS. When polymerization was performed at a low reaction time, it resulted in oligoesters with M_n_ and M_w_ values of 2022 g·mol^−1^ and 3892 g·mol^−1^, respectively (Ð = 1.9), which is similar to the OBS-L sample produced here. Similarly, Ding et al. [37] quantitatively analyzed various OBS samples in the molecular weight range of 200–1400 g·mol^−1^ obtained by different synthesis and extraction methods using ultra-high-performance polymer chromatography. Furthermore, different synthesized butylene succinate grades with a wide molecular weight range, from low-molecular-weight OBS (*M_n_* 800 g⋅mol^−1^) to high-molecular-weight PBS (*M_n_* 81,000 g⋅mol^−1^), have been reported in the literature [27]. The molecular weight differences between the samples can be attributed to multiple factors related to the synthesis conditions, including oligomerization/polymerization time, temperature, and vacuum, as well as differences in the catalysts, such as their concentration and type (efficiency).

### 3.2. Thermal and Crystallization Behavior of Oligomers

**Figure 5:**
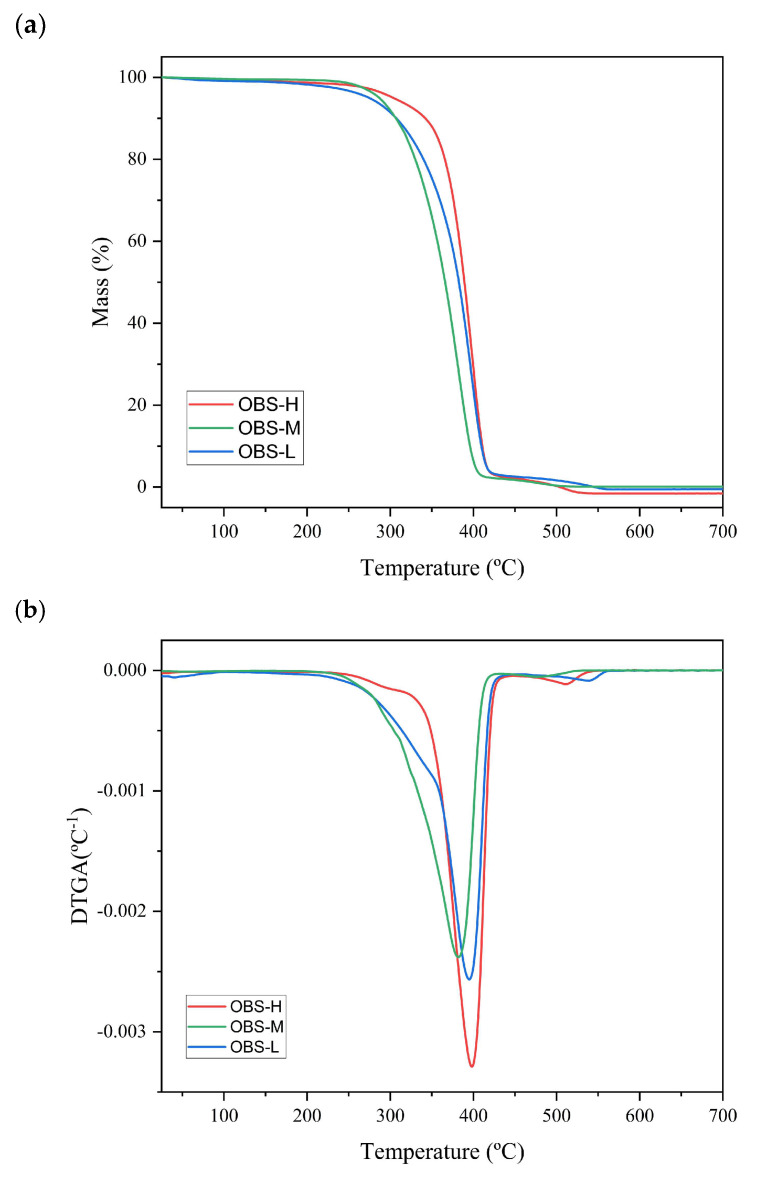
(**a**) Thermogravimetric analysis (TGA) and (**b**) first-derivative analysis (DTGA) curves of oligo(butylene succinate) with high (OBS-H), medium (OBS-M), and low molecular weights (OBS-L).

Figure 5 shows the TGA curves (Figure 5a) and their corresponding DTGA curves (Figure 5b) obtained for the three different oligomers, while Table 3 summarizes the parameters determined for each thermal degradation event. No significant weight loss occurred between 30 and 140 °C, confirming the lack of bound water and suggesting that no low-molecular-weight chains volatilized. The three oligomers exhibited different thermal stabilities depending on their molecular weights. In particular, the OBS-L, OBS-M, and OBS-H samples initiated thermal degradation at 183 °C, 223 °C, and 249 °C, respectively. Thus, low- and medium-molecular-weight oligomers showed the initiation of degradation at lower temperatures than the high-molecular-weight oligomer sample. This is consistent with the increase in thermostability with increasing molecular weight, with the highest stability being reached in longer-chain oligomers. However, their main thermal decomposition was similar in all cases, taking place in the 300–450 °C range, with a maximum degradation rate at T_peak_ of nearly 400 °C, which is consistent with the findings reported previously by Gkountela et al. [27]. The lowest T_peak_ was observed for OBS-M. In this regard, the presence of residual amounts of the metallic catalyst in the oligomers subjected to transesterification (OBS-M and OBS-H) could enhance the thermal degradation rate [41,42]. The final mass loss step at 450–550 °C can be attributed to the thermal decomposition of the organic compounds produced in the previous steps [2,43]. Therefore, as expected, the molecular weight of the oligoesters influenced their thermal stability, which is particularly relevant for the OBS-L sample because thermal degradation initiates at a temperature closer to the processing temperature of PBS (~150 °C) [44].

**Figure 6:**
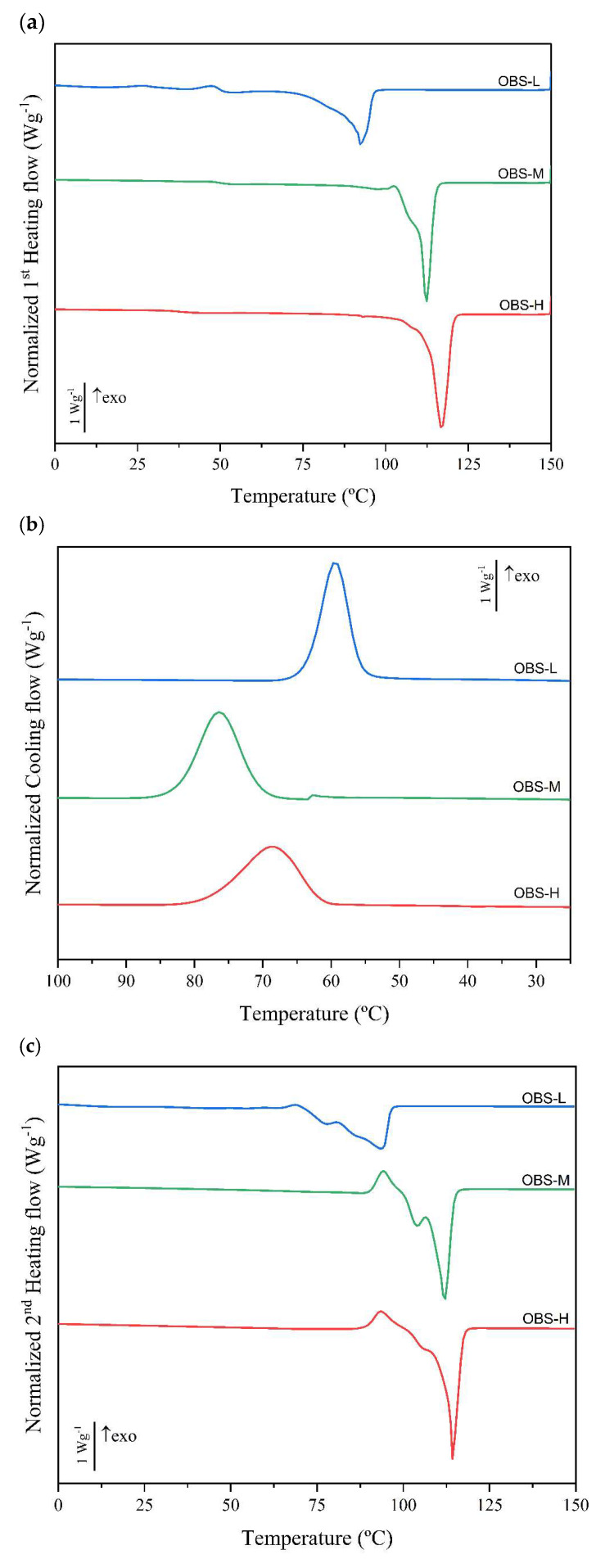
Differential scanning calorimetry (DSC) thermograms taken during the (**a**) first heating, (**b**) cooling, and (**c**) second heating of oligo(butylene succinate) with high (OBS-H), medium (OBS-M), and low molecular weights (OBS-L).

The first- and second-order phase transitions of the oligomers were analyzed using DSC. The cooling and heating thermograms obtained for the three oligomers (OBS-H, OBS-M, and OBS-L) are shown in Figure 6, whereas Table 4 summarizes the thermal transition values. During the first heating step (Figure 6a), all oligomers exhibited a low-intense thermal transition from 25 °C to 40 °C. This second-order transition has been ascribed to the glass transition corresponding to the rigid amorphous fraction (RAF) of the PBS molecules adjacent to the crystal surface, which shows a higher degree of mobility restriction, and occurs approximately at 50 °C, well above the main biopolyester’s T_g_ [2]. In the case of the OBS-H and OBS-M samples, this second T_g_ was centered at nearly 35 °C, whereas for OBS-L, it was difficult to elucidate because it overlapped with low-intensity cold crystallization peaks. In this regard, it was recently reported that RAF in PBS “devitrifies” at about 25 °C [45], which excludes any possible contribution of the RAF to the PBS melting. At higher temperatures, all oligomers melted, yielding different melting temperature (T_m_) values that increased with the degree of oligomerization and, hence, were strongly influenced by their molecular weight. In particular, T_m_ values of 94.5 °C, 112 °C, and 117.2 °C were attained for the OBS-L, OBS-M, and OBS-H samples, respectively. Melting was also observed in the form of broad peaks, indicating the presence of a wide range of crystal sizes and/or multiple phenomena of melting—recrystallization—melting in the oligomers [46,47]. In this regard, one should consider that this first thermal scan was mainly aimed at removing the thermal history of the samples, since these crystallized in the reactor under uncontrolled conditions, but it was also analyzed to better indicate the thermal response of the different oligomers.

In the cooling scans (Figure 6b), it can be observed that the three oligomers crystallized from the melt at different temperatures. In contrast to melting, the crystallization temperature (T_c_) values of the oligomers did not follow a direct trend with respect to the molecular weight. In particular, the OBS-M sample crystallized at the highest temperature (T_c_ = 76.7 °C), followed by OBS-H (T_c_ = 69.4 °C) and OBS-L (T_c_ = 59 °C). However, compared with the corresponding T_m1_ values of each oligomer, the supercooling effect directly increased with the molecular weight by 21, 27, and 37 °C, respectively, according to the reduction in molecular mobility and the subsequent limitation of the crystallization rate. A similar value for the OBS-L sample was achieved by Gkountela et al. [27], who reported a T_c_ of 53 °C for OBS with a molecular weight of 1000 g⋅mol^−1^. The crystallization behavior can also be related to the presence of particles that act as nucleating agents. In this sense, both the OBS-H and OBS-M samples were subjected to transesterification, and the remaining Ti-based particles, used as the catalyst, could act as nucleating agents, favoring their crystallization. A similar effect was reported by Ferreira et al. [48], who studied different metal catalysts and their influence on biopolyester crystallinity. The highest crystallinity was achieved with the Ti-based catalyst due to the improved lamellar structuring. In another study, Garin et al. [49] observed a higher crystallization temperature and faster rate of crystallization for PBS synthesized using a Ti-based catalyst.

In the second heating scan (Figure 6c), both the second-order transition in the 25–40 °C range and low-temperature crystallization were hardly noticed due to the recent crystallization of the biopolymer under controlled conditions (10 °C·min^−1^). The absence of aging effects favored molecular reorganization into ordered crystalline structures and disabled the formation of the so-called RAF. As observed for the first heating scan, the three oligomers cold crystallized prior to melting and then fully melted at different temperatures according to their molecular weights. Melting was also observed to occur in the form of an endothermic broad peak with two main temperatures (T_m1_ and T_m2_) or a main temperature with a shoulder due to the above-described melting—recrystallization—melting phenomena. This behavior has also been previously observed for other butylene succinate-based materials [2,50], where the defective or less stable crystalline regions melted at a lower temperature and then recrystallized as more structurally ordered crystalline domains that melted at higher temperatures. In this regard, it is worthwhile to note that the endothermic peaks (melting process) in OBS-L occurred over a significantly broader temperature range compared to the other oligomers, reaching the melting peaks of T_m1_ and T_m2_ at 79.8 °C and 94.5 °C, respectively. This is consistent with the values reported by Hariraksapitak et al. [51], who previously reported that OBS with a molecular weight of 2000 g⋅mol^−1^ melted at 78 °C, and by Gkountela et al. [27], who achieved a T_m2_ value of 96 °C for a molecular weight of 1000 g⋅mol^−1^. In the case of OBS-M, the sample yielded T_m1_ and T_m2_ values of 103.8 °C and 111.7 °C, respectively. Similar values of T_m2_ were achieved by Palamidi et al. [52] for an OBS of 4000 g⋅mol^−1^. In contrast, OBS-H showed a single melting peak (T_m_ = 114.3 °C) with a shoulder, similar to that of PBS [2].

Finally, the crystallinity percentage or *X_c_* of the synthesized oligomer samples (OBS-H, OBS-M, and OBS-L) was determined from their normalized enthalpies of melting. To this end, different reference values of *ΔH°_m_* reported for PBS were employed. The three oligomer samples presented *ΔH_m_* values in the 70–80 J/g range, without significant differences (*p* > 0.05) between them, but slightly higher for the OBS-L. Table 4 also shows the *X_c_* values based on the reported *ΔH°_m_* values of 110.3 J/g [27] and 102 J/g [28], whose values ranged from approximately 64% to 78%. However, taking *ΔH°_m_* as 200 J/g [30,31] resulted in a crystallinity degree in the range of 35–40%. These values are similar to those reported in our previous study for fully bio-based PBS (*X_c_* = 36.2 ± 1.7%) [16]. Similarly, Gkountela et al. [27] reached crystallinity percentages of 73% and 67% for OBS with 1150 g⋅mol^−1^ and 1400 g⋅mol^−1^, respectively, using *ΔH°_m_* values of 110.3 J/g. Due to the large differences in the *X_c_* values and the lack of any specific *ΔH°_m_* reference for OBS, the crystallinity of the oligomers was elucidated by X-ray diffraction. The DSC results suggest that the molecular weight of OBS has a minor effect on its crystallinity.

### 3.3. XRD Crystallinity Analysis of Oligomers

**Figure 7:**
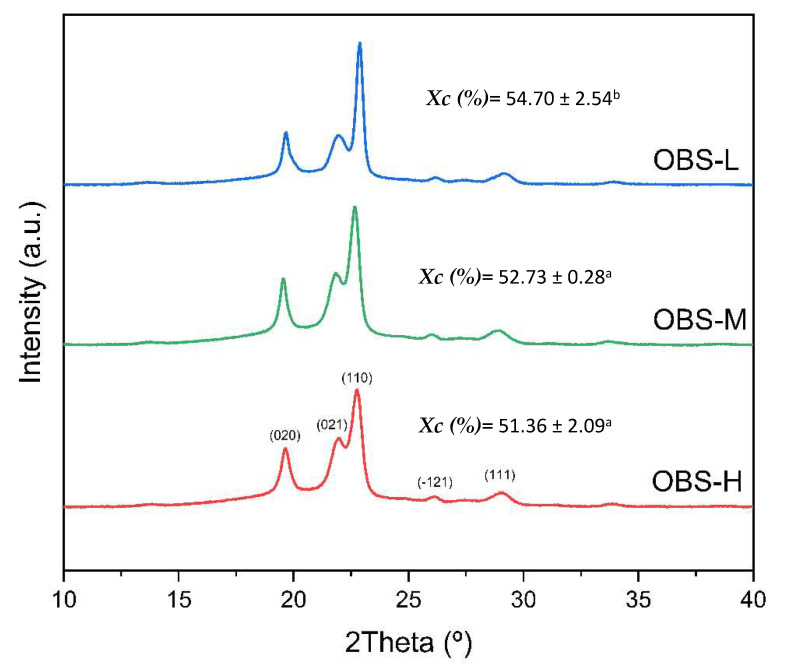
X-ray diffractograms with peak assignments of oligo(butylene succinate) with high (OBS-H), medium (OBS-M), and low molecular weights (OBS-L). ^a–b^ Different superscripts in the percentage of crystallinity (*χ*_c_) values indicate significant differences among the samples (*p* < 0.05).

The oligomer powders were further subjected to crystallinity analysis using X-ray diffraction. Figure 7 shows the diffractograms of the three oligomer samples (OBS-H, OBS-M, and OBS-L) with peak assignments for the different crystallographic planes. All the OBS samples showed the five main characteristic diffraction peaks previously reported for PBS, located at around 19.5°, 22.0°, 22.5°, 25.9°, and 28.8°, which were assigned to the (020), (021), (110), (−121), and (111) planes, respectively [45,53,54]. Therefore, OBS exhibited a diffraction pattern similar to that of fully bio-based PBS, corresponding to the monoclinic crystal lattice of the homopolyester α-form [16]. As shown in the diffractograms, the ratio of crystalline to amorphous areas of the diffraction peaks resulted in samples with slight differences in crystallinity. In particular, the *X_c_* values exhibited an inverse relationship with the molecular weight, increasing progressively from 51% (OBS-H) to nearly 55% (OBS-L). The slightly but still significantly different (*p* < 0.05) *X_c_* value attained for the OBS-L sample suggests that crystallinity is promoted due to the chain mobility increases as a result of the molecular weight reduction. Thus, shorter oligomer chains can more readily adopt conformations conducive to crystalline packing, while reduced viscosity in the melt state facilitates molecular reorganization during crystallization. In any case, the *X_c_* values attained for the oligomers are very close to that reported for fully bio-based PBS (41.4%) [16] and intermediate to those described above during the DSC analysis, depending on the different *ΔH°_m_* values reported in the literature for PBS.

**Figure 8:**
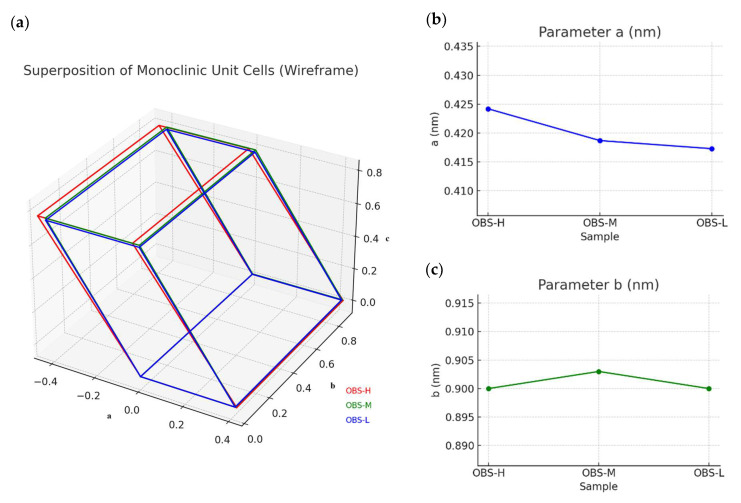

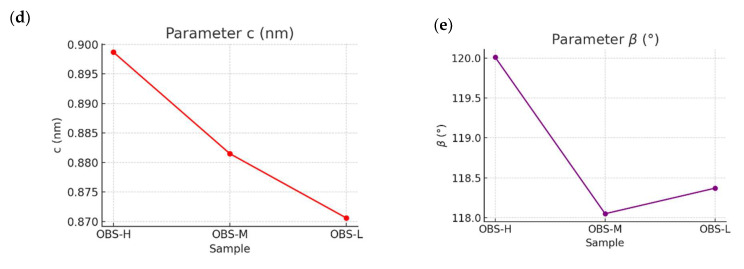
(**a**) Monoclinic unit cells of oligo(butylene succinate) with high (OBS-H), medium (OBS-M), and low (OBS-L) molecular weights with the evolution of their (**b**) *a*, (**c**) *b*, and (**d**) *c* lattice dimensions, and (**e**) *β* angle parameter.

In terms of the unit cell, Figure 8 shows the monoclinic unit cell of the oligomers (Figure 8a) and the values of the *a*, *b*, *c*, and *β* angle parameters for each oligomer with different molecular weights. Thus, the monoclinic unit cell lattice parameters showed the following systematic variations: *a*-axis systematically decreased with decreasing molecular weight (0.4259 nm → 0.4173 nm), indicating tighter lateral packing of chains in the crystal lattice and suggesting more efficient molecular organization in shorter chains (Figure 8b); *b*-axis remained nearly constant across all samples (~0.900 nm), which indicates preservation of fundamental chain conformation (Figure 8c); *c*-axis progressively decreased with decreasing molecular weight (0.9103 nm → 0.8706 nm), reflecting a more compact packing along the crystallographic *c*-direction due to enhanced interchain interactions among the oligomer chains (Figure 8d); *β* angle exhibited a slight variation (119.09° → 118.37°), maintaining the monoclinic crystal system with minor adjustments that accommodate optimal packing (Figure 8e). This evolution was further supported by a comparison with the unit cell parameters of fully bio-based PBS, which presented values of *a* = 0.531 nm, *b* = 0.900 nm, *c* = 1.141 nm, and β = 125.48° [16]. Therefore, this comprehensive crystallinity analysis revealed that the molecular weight reduction in OBS led to systematic changes in the crystalline structure, favoring crystal organization with denser packing. The preservation of the monoclinic system across all samples also indicates the retention of the fundamental PBS crystal structure, while subtle adjustments in the unit cell lattice parameter could accommodate the optimal packing of the shorter chains.

To the best of our knowledge, the lattice dimensions of either OBS or PBS have not been previously analyzed as a function of molecular weight. However, Righetti et al. [55] reported the temperature evolution of the *a*, *b*, and *c* lattice dimensions of a PBS grade of 2.8⋅10^4^ g⋅mol^−1^ by applying isotherms in the 100–115 °C range in WAXS using synchrotron radiation. The authors observed that the cell dimensions, which were also calculated from the angular position of the five most intense peaks through a least-squares fitting procedure, did not increase regularly due to mere thermal expansion. The three lattice values remained approximately constant in the temperature range 105–109 °C, then increased slowly between 109 and 111 °C, and finally achieved a more significant enlargement up to 115 °C. Therefore, it was concluded that the most stable crystal structure was developed in the temperature interval 105–111 °C due to the growth of the crystal organization at temperatures prior to melting. This observation, in combination with the effect of molecular weight reported herein, indicates that the crystal dimensions of OBS are affected by both the synthesis conditions of the oligomers and the thermal exposure of the materials.

## 4. Conclusions

Proper control and extension of the multi-step polycondensation process conditions applied for the synthesis of fully bio-based PBS successfully allowed herein the obtaining of oligomers with varying molecular weights. Thus, three oligomers with M_w_ values of 2050 g·mol^−1^ (OBS-L), 16,150 g·mol^−1^ (OBS-M), and 33,147 g·mol^−1^ (OBS-H), and Ð values in the 1.7–1.8 range were effectively produced. The molecular weights of these oligomers were approximately 58, 7, and 3.5 times lower than the fully bio-based homopolyester; thus, being the OBS-M and OBS-H samples also within the range of low-molecular-weight PBS. The thermal degradation stability of the synthesized oligomers increased slightly with molecular weight, whereas the two oligomers with the highest molecular weight yielded thermal transitions similar to those of PBS. The crystallinity of OBS also decreased when the molecular weight increased. Interestingly, the molecular weight evolution of the lattice parameters suggested that the shorter chains of OBS-L favored crystal organization, resulting in a crystal unit cell with denser packing. Future studies will focus on using these oligomers as novel additives in biopolyester-based packaging materials and particularly investigate their incorporation into PBS matrices to improve both the processability and properties of this succinic acid derived biopolyester.

## Figures and Tables

**Table 1:** Chemical shifts (δ in ppm) and integrals before and after derivatization with trifluoroacetic anhydride of proton signals for oligo(butylene succinate) with high (OBS-H), medium (OBS-M), and low molecular weights (OBS-L).

**Sample**	**CH_2_OOC**	**CH_2_OH**	**CH_2_OTFA**	**CH_2_COO**	**CH_2_COTFA**	**CH_2_CH_2_O**
	**Chemical Shifts**					
	δ = 4.12	δ = 3.67	δ = 4.40	δ = 2.63	δ = 2.95	δ = 1.70
	**Integrals before Derivatization**					
**OBS-H**	572.2	2.0	-	576.0	-	574.0
**OBS-M**	110.7	2.0	-	110.9	-	112.5
**OBS-L**	8.1	2.0	-	6.6	-	10.1
	**Integrals after derivatization**					
**OBS-H**	573.0	-	2.0	570.0	5.6	574.5
**OBS-M**	110.5	-	2.0	109.3	1.3	112.0
**OBS-L**	8.1	-	2.0	6.4	0.3	10.1

**Table 2:** Number- and weight-average molecular weights (M_n_ and M_w_) and dispersity index (Ð) of the oligo(butylene succinate) with high (OBS-H), medium (OBS-M), and low molecular weights (OBS-L).

Sample	M_n_ (g·mol^−1^) ^a^	M_n_ (g·mol^−1^) ^b^	M_w_ (g·mol^−1^) ^b^	Ð
OBS-H	12,900	18,650	33,147	1.7
OBS-M	5950	8700	16,150	1.9
OBS-L	900	1100	2050	1.8

^a^ Determined by proton nuclear magnetic resonance (^1^H-NMR). ^b^ Determined by gel permeation chromatography (GPC).

**Table 3:** Temperatures at the onset of degradation (T_onset_), maximum degradation rate (T_peak_), and end of degradation (T_end_) of oligo (butylene succinate) with high (OBS-H), medium (OBS-M), and low molecular weights (OBS-L).

Oligomer	25–450 °C		
	T_onset_ (°C)	T_peak_ (°C)	T_end_ (°C)
OBS-H	249 ± 4 ^c^	398 ± 0 ^b^	423 ± 3 ^b^
OBS-M	223 ± 1 ^b^	379± 2 ^a^	410 ± 1 ^a^
OBS-L	183 ± 3 ^a^	396 ± 1 ^b^	420 ± 1 ^b^

^a–c^ Different superscripts in the same column indicate significant differences among samples (*p <* 0.05).

**Table 4:** Crystallization temperatures (T_c_), enthalpy of crystallization (∆H_c_), cold crystallization temperature (T_cc_), two main melting temperatures (T_m1_, T_m2_), enthalpy of melting (∆H_m_), and percentage of crystallinity (*χ*_c_) of the oligo(butylene succinate) with high (OBS-H), medium (OBS-M) and low (OBS-L) molecular weight.

Oligomer	Cooling		Second Heating						
	T_c_ (°C)	∆H_c_ (J/g)	T_cc_ (°C)	T_m1_ (°C)	T_m2_ (°C)	∆H_m_ (J/g)	X_c_ (%) (*ΔH°_m_* = 110.3 J/g)	X_c_ (%) (*ΔH°_m_* = 102 J/g)	X_c_ (%) (*ΔH°_m_* = 200 J/g)
OBS-H	69.4 ± 1.2 ^b^	73.0 ± 2.6 ^a^	93.8 ± 0.4 ^c^	106.62 ± 0.3 ^c^	114.3 ± 0.1 ^c^	73.5 ± 0.7 ^a^	66.6 ± 0.6 ^a^	72.0 ± 0.6 ^a^	36.7 ± 0.3 ^a^
OBS-M	76.7 ± 0.4 ^c^	83.4 ± 2.9 ^a^	94.3 ± 0.1 ^b^	103.77 ± 0.4 ^b^	111.7 ± 0.2 ^b^	70.8 ± 3.3 ^a^	64.2 ± 2.9 ^a^	69.4 ± 3.2 ^a^	35.4 ± 1.6 ^a^
OBS-L	59.0 ± 0.5 ^a^	68.6 ± 8.7 ^a^	71.1 ± 0.9 ^a^	79.81 ± 0.8 ^a^	95.6 ± 0.7 ^a^	78.9 ± 4.5 ^a^	71.6 ± 4.1 ^a^	77.4 ± 4.4 ^a^	39.4 ± 2.2 ^a^

^a–c^ Different superscripts in the same column indicate significant differences among the samples (*p <* 0.05). Melting enthalpy of a fully crystalline poly(butylene succinate) (PBS) (*ΔH°_m_*): 110.3 J/g [27], 102 J/g [28], and 200 J/g [30,31].

## Data Availability

Data are contained within the article.
